# Members of the *RAD52* Epistasis Group Contribute to Mitochondrial Homologous Recombination and Double-Strand Break Repair in *Saccharomyces cerevisiae*

**DOI:** 10.1371/journal.pgen.1005664

**Published:** 2015-11-05

**Authors:** Alexis Stein, Lidza Kalifa, Elaine A. Sia

**Affiliations:** Department of Biology, University of Rochester, Rochester, New York, United States of America; Duke University, UNITED STATES

## Abstract

Mitochondria contain an independently maintained genome that encodes several proteins required for cellular respiration. Deletions in the mitochondrial genome have been identified that cause several maternally inherited diseases and are associated with certain cancers and neurological disorders. The majority of these deletions in human cells are flanked by short, repetitive sequences, suggesting that these deletions may result from recombination events. Our current understanding of the maintenance and repair of mtDNA is quite limited compared to our understanding of similar events in the nucleus. Many nuclear DNA repair proteins are now known to also localize to mitochondria, but their function and the mechanism of their action remain largely unknown. This study investigated the contribution of the nuclear double-strand break repair (DSBR) proteins Rad51p, Rad52p and Rad59p in mtDNA repair. We have determined that both Rad51p and Rad59p are localized to the matrix of the mitochondria and that Rad51p binds directly to mitochondrial DNA. In addition, a mitochondrially-targeted restriction endonuclease (mtLS-*Kpn*I) was used to produce a unique double-strand break (DSB) in the mitochondrial genome, which allowed direct analysis of DSB repair *in vivo* in *Saccharomyces cerevisiae*. We find that loss of these three proteins significantly decreases the rate of spontaneous deletion events and the loss of Rad51p and Rad59p impairs the repair of induced mtDNA DSBs.

## Author Summary

Mitochondria are the powerhouse of the cell, providing energy in the form of ATP. Several proteins required for the production of ATP are encoded in the mitochondrial genome, which is maintained independently from the nuclear genome. Mutations in mitochondrial DNA are responsible for several inherited diseases and are associated with certain cancers, neurological disorders, and aging. In human mtDNA, 90% of deletions, a specific type of mutation, are flanked by repetitive sequences. Deletions in this context are generally produced through homologous recombination, specifically single-strand annealing. For the first time, in this study we show that the proteins involved in nuclear homologous recombination, Rad51p, Rad52p, and Rad59p, also contribute to the formation of mitochondrial deletions. Our data also suggest a novel mitochondrial-specific role for Rad51p in the generation of this type of deletion. Further study of these repair pathways allows us to garner a better understanding of these processes that are involved in disease pathologies and aging.

## Introduction

Mitochondria are dynamic organelles that function as the primary source of ATP. Mitochondria contain an autonomously replicating genome that is packaged into DNA-protein structures called nucleoids. This multi-copy genome encodes subunits of electron transport chain (ETC) complexes and the rRNAs and tRNAs that are specifically required for the expression of these proteins. The number and types of proteins encoded by the mitochondrial genome are highly conserved among eukaryotes, even though the overall size and structure of the genome varies widely between species. The remaining proteins required for the ETC, as well as those required for maintenance and repair of the mitochondrial genome are encoded in the nucleus and imported into mitochondria from the cytoplasm.

Stability of the mitochondrial genome is required for the survival of most eukaryotic cells. Mutations in the mitochondrial genome cause metabolic defects that underlie multiple diseases, both inherited and spontaneous [1–6]. Accumulation of both point mutations and deletions in mtDNA are also proposed to contribute to the normal aging process [7]. Consistent with this hypothesis, transgenic mice expressing a proofreading-defective Pol γ, the replicative mitochondrial polymerase, display accelerated aging phenotypes due to the accumulation of somatic mtDNA mutations [8]. Despite its profound importance to metabolic control and cell maintenance, the mechanisms of mitochondrial mutagenesis and repair are poorly understood, particularly double-strand break repair (DSBR) [9]. Though homologous recombination (HR) in yeast, plant and mammalian mtDNA has been reported [10–15], few specific proteins involved in mitochondrial recombination have been identified [16–22]. For those proteins that have been implicated in mitochondrial HR, the inability to generate and visualize specific, controlled mitochondrial double-strand breaks has precluded a direct analysis of their role in DSBR.

**Fig 1:**
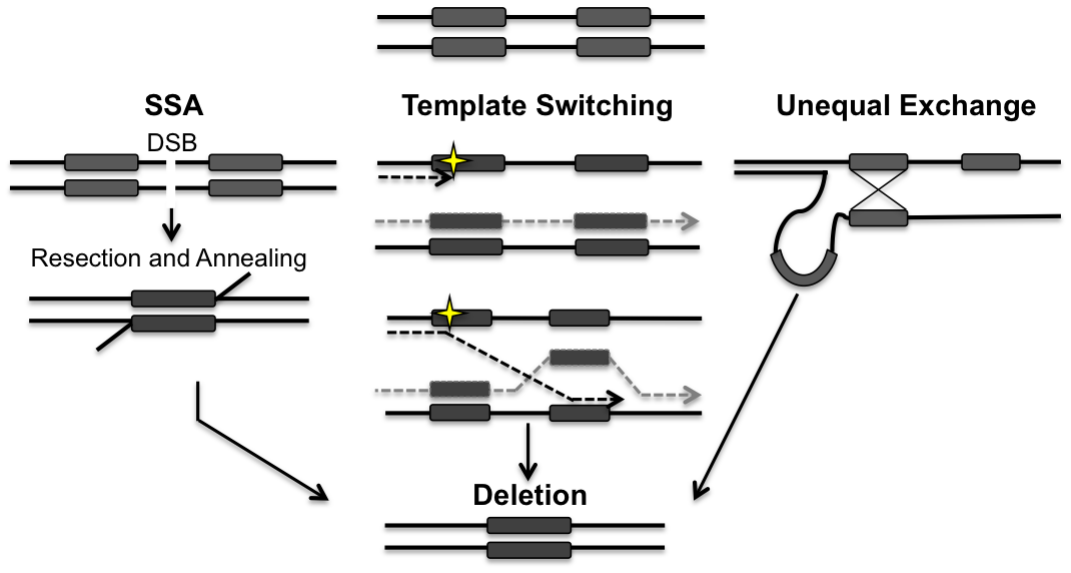
Models for the generation of deletions between directly repeated sequences. Repair of a double-strand break (DSB) located between directly repeated sequences (grey boxes) can result in the deletion of one of the repeats and all of the intervening sequence. 5’ to 3’ resection at the DSB reveals the direct repeats. Rad52p alone or in conjunction with Rad59p, promotes the annealing of the repeats. Template switching can occur if a lesion (yellow star) is encountered during replication. At a stalled fork, the nascent strand may invade at the incorrect repeat, leading to the generation of a deletion. Unequal exchange can occur when HR occurs between misaligned repeats leading to a deletion. Both template switching and unequal exchange can happen intramolecularly or intermolecularly. Arrowheads indicate 3’ end.

Members of the *RAD52* epistasis group are essential for HR in the nucleus. Rad51p, a RecA homologue, forms a nucleoprotein filament that promotes the homologous pairing and strand exchange required for DSBR, synthesis dependent strand annealing and break induced replication [23–25]. Rad52p is required for almost all forms of HR in yeast, by promoting the exchange of RPA for Rad51p on the single-stranded ends that are generated at DSBs following resection [26]. HR is often thought of as an error-free repair pathway but there are several HR mechanisms that can lead to the generation of deletions between repetitive elements. For example single strand annealing (SSA), which is highly dependent on Rad52p and Rad59p, is an error-prone recombination pathway that occurs when a DSB or a lesion that results in a DSB arises between two repetitive sequences [27]. The annealing of these repetitive sequences after resection leads to the deletion of one repeat and the intervening sequence (Fig 1) [26,28]. A DSB that arises within a repeat can also generate a deletion if the repetitive sequences are misaligned during unequal crossing over (Fig 1) [29]. Deletions may also arise between repeated sequences due to errors in replication such as slippage or template switching (Fig 1) [30].

Deletions have been observed in yeast, plants, flies, and mammalian mtDNA [10,11,31,32], and often these deletions involve sequences originally flanked by direct repeats. For example, in human cells nearly 90% of deletions in mtDNA are flanked by either perfect or imperfect repeats. This suggests that recombination is a possible mechanism for the generation of mtDNA deletions, but the exact mechanisms and proteins involved are currently unknown. [33,34].

Recent studies have localized members of the *RAD52* epistasis group to mitochondria. In plants, a mitochondrial-specific isoform of Rad52p has been identified, and demonstrated to promote annealing of complementary DNA sequences *in vitro*, consistent with a role in recombination in mitochondria [35]. In human cell lines, Rad51, and two of its paralogs Rad51C and XRCC3 were found to localize to mitochondria and promote mtDNA replication upon oxidative stress [36,37]. These studies strongly suggest that at least a subset of the nuclear recombination proteins can localize to mitochondria and that once there, they may facilitate processes that require homologous sequences such as HR. Mammalian systems do not lend themselves to mechanistic studies because these organisms require intact mtDNA molecules to survive. In addition, it is not currently possible to introduce exogenous DNA reporters *in vivo* in these systems. *Saccharomyces cerevisiae* is an ideal model system for these studies due to the fact that these repair proteins are highly conserved, mtDNA is not required for cell survival, and it is possible to introduce exogenous reporter constructs directly into the mitochondrial genome.

We previously developed a reporter system for quantitatively measuring the occurrence of direct repeat mediated deletions (DRMD) in mtDNA [38–40]. This reporter introduces a unique *Kpn*I restriction recognition site between direct repeats. By expressing mitochondrially-targeted *Kpn*I, induction of a directed mtDNA DSB can be precisely controlled. In the work presented here, we use a modified version of this endonuclease fusion to demonstrate that loss Rad51p, Rad52p and Rad59p significantly decreases the generation of both spontaneous and DSB induced mitochondrial DRMDs. Improved control of DSB induction has also allowed us to study the kinetics of mtDNA DSBR using both genetic and molecular methods. Southern blot analysis confirms that loss of these proteins leads to delays in break processing and/or generation of deletion products. The localization of Rad51p and Rad59p to yeast mitochondria argue that these effects are direct. Analysis of double mutants indicates that the contribution of these proteins to the repair process differs between the mitochondrial and the nuclear genomes.

## Results

### Rad51p and Rad59p localize to the mitochondrial matrix

**Fig 2:**
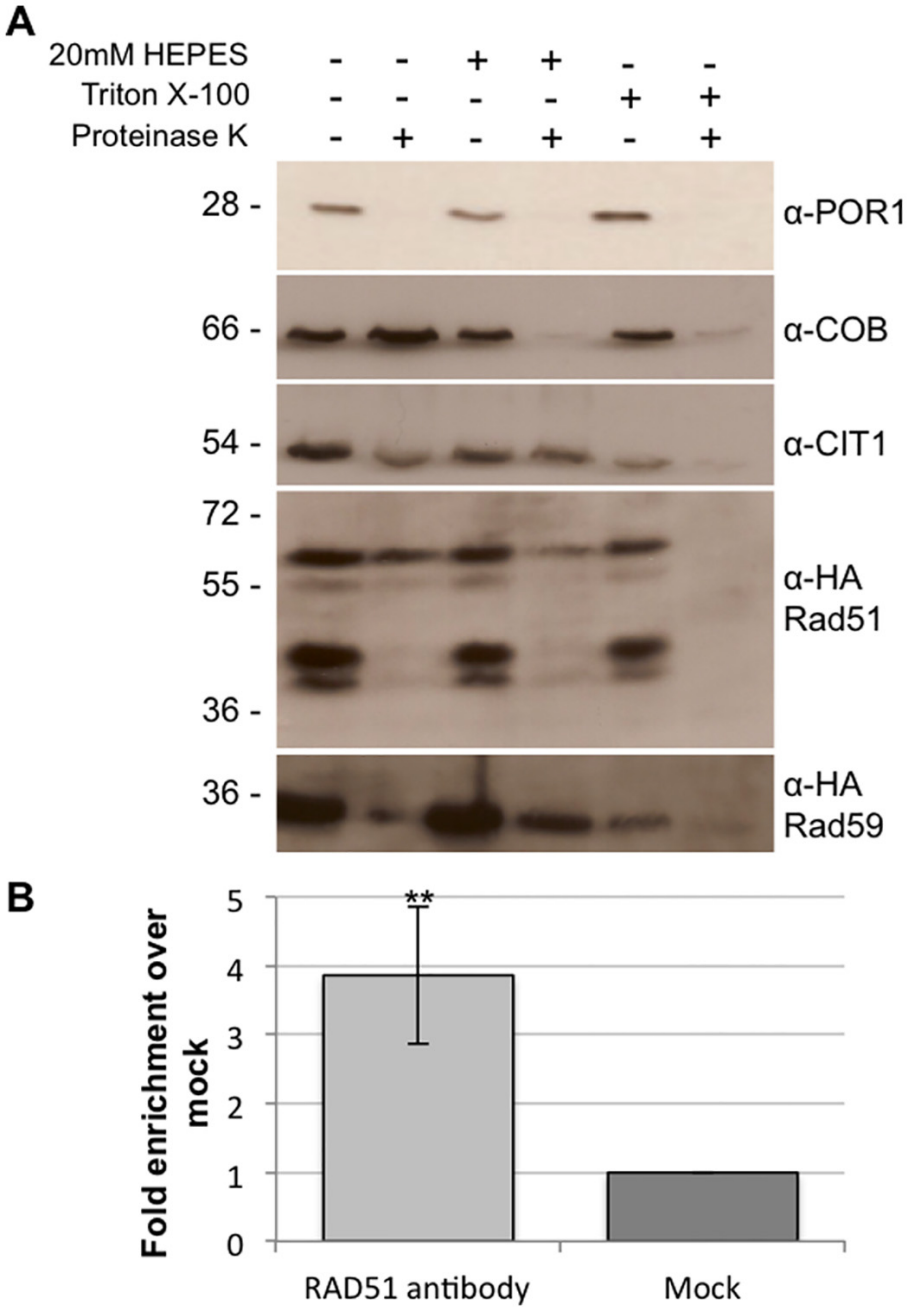
Rad51p and Rad59p localize to the mitochondrial matrix. (A) Intact mitochondria, mitoplasts, or Triton X-100 lysed mitochondria were treated with proteinase K and subjected to immunoblot analysis with the indicated antibodies. Por1p, outer membrane protein; Cob, intermembrane space protein; Cit1p, matrix protein. (B) Rad51p physically interacts with mtDNA. DNA samples (3 biological replicates) were immunoprecipitated with antibodies against endogenous Rad51p or V5 epitope tag (mock). Samples were evaluated by qPCR and changes in fold enrichment were compared to the mock IP. Error bar indicates SD. Asterisks indicate significant differences between the Rad51p IP and the mock IP (** = *p* ≤ 0.01).

Previous studies have shown that Rad51, Rad51C and XRCC3 are found in the mitochondria of human cells [36]. In order to determine if this dual localization of Rad51 to both the nucleus and mitochondria is conserved in yeast, mitochondria were purified from cells expressing a C-terminal Rad51-HA tag expressed from the endogenous locus. We were able to detect at least four Rad51-HA specific bands in whole cell extracts and associated with the mitochondrial fraction (Fig 2A). These bands are specific to the strain expressing the Rad51-HA as they cannot be detected in whole cell extracts or mitochondrial extracts from an untagged control strain (S3 Fig). In order to determine the precise mitochondrial localization a protease protection assay was performed. Isolated mitochondria were subjected to proteinase K treatment in the presence or absence of 20mM Hepes, pH 7.4 to remove the outer mitochondrial membrane, or Triton X-100 to rupture both membranes. The inter membrane space protein, cytochrome b (Cob), is sensitive to digestion when the mitochondria are treated with 20mM Hepes (Fig 2A). The ~ 55 kDa band of Rad51p is also sensitive to digestion when the mitochondria are treated with 20mM Hepes suggesting this isoform is also found in the inter membrane space (Fig 2A, lanes 1 and 2). It is possible this isoform is also found in the matrix but is not detectable with our current system. Citrate synthase (Cit1p), a matrix protein (Fig 2A) and the ~ 60 kDa band of Rad51 remains protected from proteinase K unless treated with Triton X-100, confirming Rad51p’s localization in the matrix of the mitochondria in yeast (Fig 2A, lane 4). The specific modification(s) responsible for the larger Rad51p mitochondrial isoform(s) is currently unknown. Mitochondria were also purified in the same manner from cells expressing Rad59-HA from the endogenous locus. A protease protection assay confirmed the presence of Rad59 in the matrix of the mitochondria (Fig 2A and S3 Fig).

In order to further confirm the mitochondrial localization of Rad51p and to determine if it binds directly to mtDNA, chromatin immunoprecipitation (ChIP) was performed using an antibody to the native untagged Rad51 protein. Using primers that anneal to a region previously demonstrated to be near a recombination hotspot in the yeast mitochondrial genome, we were able to detect a significant 4-fold (*p* = 0.008) increase in mtDNA signal in the Rad51p IP compared to the mock IP (Fig 2B) [41]. The western blot and ChIP data together clearly demonstrate the Rad51p is localized to the mitochondria of *S*. *cerevisiae*.

### Rad51p, Rad52p, and Rad59p promote spontaneous mitochondrial deletions

Rad51p, Rad52p, and Rad59p participate in spontaneous DRMD events and the repair of induced DSBs in the nucleus [26]. To determine whether they also contribute to spontaneous mitochondrial DRMD, we generated deletions of these genes both individually and in combination, in yeast strains containing genetic reporters for nuclear and mitochondrial DRMD events [38–40].

**Fig 3:**
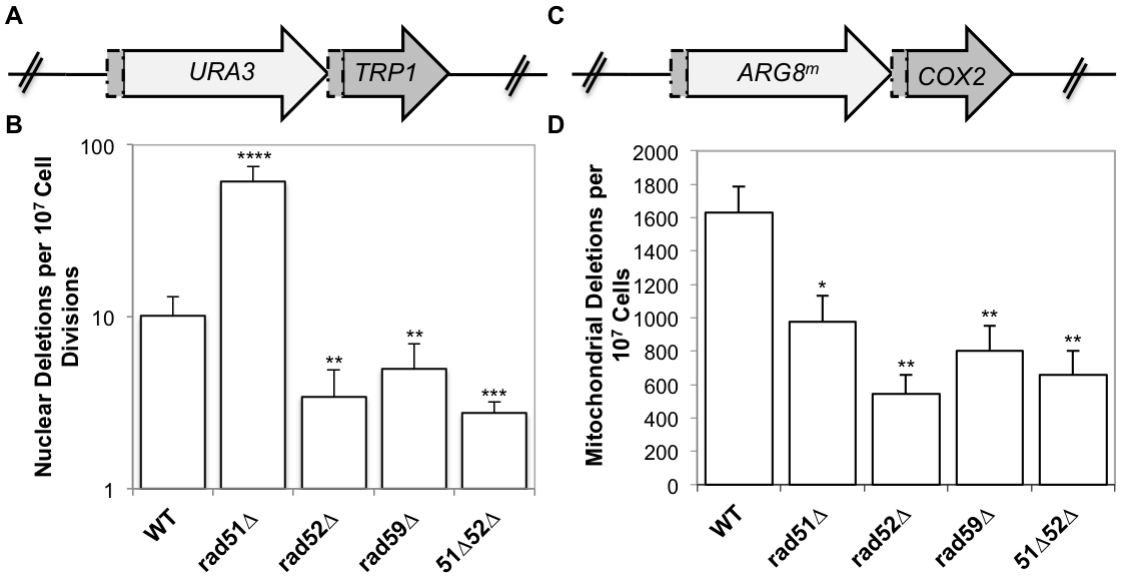
Spontaneous nuclear and mitochondrial repeat mediated deletions. (A) The nuclear DRMD reporter. The *URA3* gene was inserted 99 bp into the *TRP1* sequence, and is followed directly by the entire *TRP1* gene lacking the start codon, resulting in a 96 bp direct repeat flanking the *URA3* marker. Spontaneous deletions are selected on medium lacking tryptophan, and rates were determined using the method of the median. (B) The mitochondrial DRMD reporter. The *ARG8* ^*m*^ gene was inserted 99 bp into the *COX2* sequence, and is followed directly by the entire *COX2* gene lacking the start codon, resulting in a 96 bp direct repeat flanking the *ARG8* ^*m*^ marker. Spontaneous deletions are selected on medium containing glycerol as the sole carbon source, and rates were determined using the method of the median. (C) Average rates of nuclear DRMD. (D) Average rates of mitochondrial DRMD. Error bars indicate SD. Asterisks indicate significant differences between the mutant and wild-type rates (* = *p* ≤ 0.05, ** = *p* ≤ 0.01, *** = *p* ≤ 0.001, **** = *p* ≤ 0.0001).

The mitochondrial reporter consists of the *ARG8* ^*m*^ gene, a mitochondrial derivative of the nuclear *ARG8* gene that has been recoded to reflect the codon usage and bias of a mitochondrial gene [42]. The *ARG8* ^*m*^ gene is inserted 99 bp into the *COX2* gene followed by the entire *COX2* gene lacking the start codon (Fig 3B). This generates 96 bp of directly repeated sequence flanking *ARG8* ^*m*^. Strains that contain an intact reporter are phenotypically Arg^+^ and respiration deficient. When a mitochondrial DRMD event occurs, the strain becomes respiration proficient and will become Arg^-^ following sorting to homoplasmy, which occurs rapidly in yeast. The nuclear reporter is similarly constructed, with the *URA3* gene inserted 99 bp into the *TRP1* gene, followed by the entire *TRP1* gene, generating 96bp of directly repeated sequence (Fig 3A) [39]. Strains with an intact reporter are phenotypically Ura^+^ and Trp^-^. If a nuclear DRMD event occurs, the strain becomes Ura^-^ and Trp^+^. Plating of cells on the appropriate media allows selection of both mitochondrial and nuclear deletions from the same culture.

As we have previously shown, the mitochondrial deletion rate is approximately160-fold greater than the nuclear rate for similar repeats in wild-type cells. It is important to note that nuclear and mitochondrial rates should not be compared directly, since the multicopy nature of the mitochondrial genome, and its subsequent replication and segregation into daughter cells will impact the rate. Rates within each compartment are compared between wild-type and mutant strains. Deletion of *RAD52* or *RAD59* resulted in similar decreases of both nuclear and mitochondrial DRMDs (Fig 3C and 3D). Spontaneous nuclear and mitochondrial DRMDs decreased 3-fold in the *rad52*-Δ strain (*p* = 0.001, in each case) and 2-fold in the *rad59*-Δ strain (nuclear *p* = 0.001, mitochondrial *p* = 0.002) relative to wild-type. This suggests that the contribution of Rad52p and Rad59p to the generation of spontaneous deletions is equally as important in both the nucleus and mitochondria. In contrast, *RAD51* deletion resulted in different nuclear and mitochondrial phenotypes, with a 6-fold increase in nuclear DRMD (*p* = 2 x 10^-8^) and an approximately 2-fold decrease in mitochondrial DRMD (*p* = 0.01) (Fig 3C and 3D). The nuclear spontaneous DRMD results for this strain are consistent with the current model of SSA, however, the decrease measured for mitochondrial DRMD is not. According to the current nuclear model, SSA is dependent on Rad52p and Rad59p and independent of Rad51p. Deletions generated by SSA increase in the absence of accurate repair mediated by Rad51p [28,43]. Thus, the observed decreases in nuclear DRMD events in the *rad52-*Δ, *rad59-*Δ, and *rad51-*Δ *rad52-*Δ strains, and the increase in nuclear DRMD events in *rad51-*Δ strains were as expected [27,28]. The decrease in mitochondrial DRMD events observed in the *rad51* null mutants indicates that in contrast to the nuclear DRMD events, spontaneous mitochondrial DRMDs are at least partially dependent on Rad51p, suggesting a unique functional role for Rad51p in mitochondria.

To confirm that the decreases in mitochondrial DRMD rates in the *rad* mutants are due to specific deficiencies in mitochondrial pathways leading to deletions, and not due to general mitochondrial genome instability, the spontaneous frequency of *petites* was measured in each of the above mutants. Spontaneously arising *petite* colonies (*ρ* ^-^) are non-respiring, usually due to large-scale deletions and rearrangements of mtDNA, and do not grow on non-fermentable carbon sources such as glycerol [10]. The mechanism by which these deletions and rearrangements occur has not been defined, although homologous recombination has been proposed [10]. None of the deletions we tested exhibited any significant change from wild-type in the frequency of respiration loss (S2 Table). This suggests that the phenotypes we observe do not result from a general increase in mtDNA instability, and these proteins are not involved in the mechanism that generates spontaneous *petite* colonies.

### Specific mitochondrial double-strand breaks increase the frequency of mtDNA deletions

**Fig 4:**
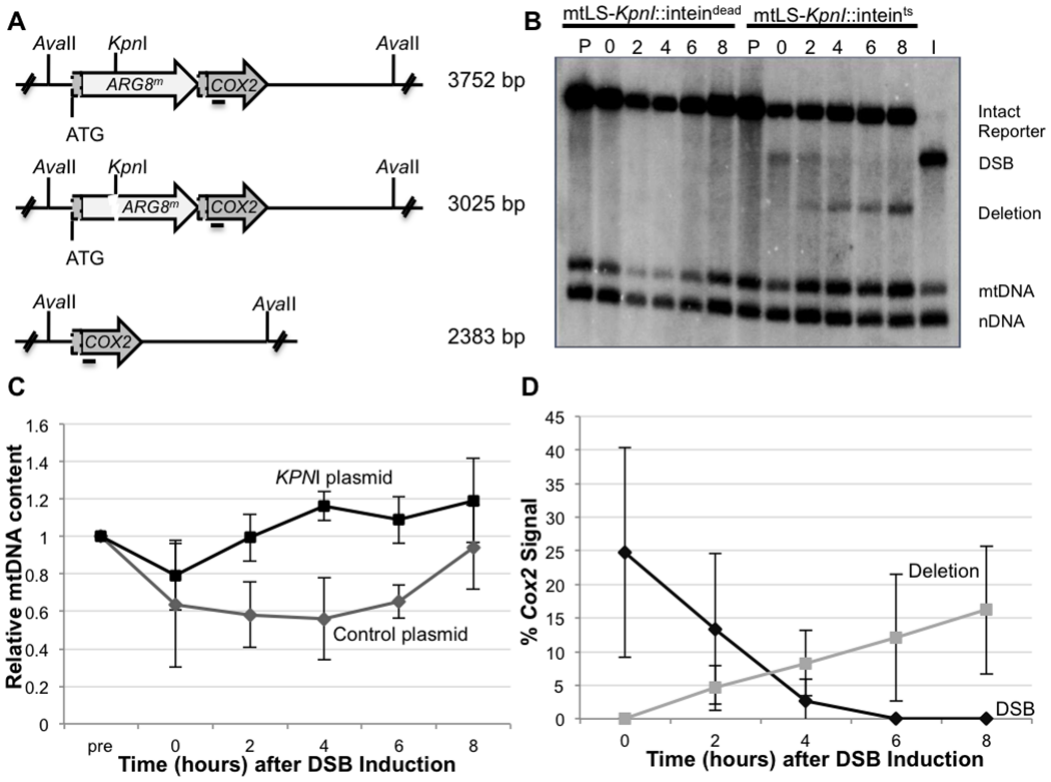
Inducing a specific mitochondrial DSB. (A) The mitochondrial DRMD reporter, with restriction recognition sequences indicated. Horizontal line beneath *COX2* indicates where the *COX2* probe anneals. (B) Representative Southern blot of *Ava*II-digested DNA extracted from EAS748 containing pEAS115 (mtLS-*Kpn*I-intein^dead^) or pEAS114 (mtLS-*Kpn*I-intein^ts^). Both strains were grown in SRaffinose-Arg-Ura until OD_600_ = 0.150. Galactose was added to a final concentration of 2%, and the strains were shifted to 20°C to induce the either mtLS-*Kpn*I-intein^ts^ or mtLS-*Kpn*I-intein^dead^. Genomic DNA in lanes labeled P are from preinduced cultures. Cultures were incubated for 16–18 hours at 20°C and samples were taken for the time 0 timepoint. The cultures then were shifted to 30°C to allow repair. Lane I contains DNA from the t = 0 timepoint of the mtLS-*Kpn*I-intein^ts^-containing strain digested *in vitro* with *Kpn*I and *Ava*II to demonstrate the migration of DNA with a DSB. The 21S rRNA gene was probed to detect total mtDNA. The 25S rRNA gene was probed to detect total nuclear DNA. (C) Relative mtDNA content was determined by taking the ratio of the 21S rRNA signal and the 25S rRNA signal, then normalizing to the t = 0 sample for each strain. (D) The intensity of the *COX2*-hybridizing bands were measured using Image Lab software (www.biorad.com) The proportions of the total *COX2* signal present in the DSB and deletion products were calculated as a percent of the total *COX2* signal.

The *ARG8* ^*m*^ sequence used in our mitochondrial reporter contains a unique *Kpn*I restriction site. Cleavage at this site generates a specific DSB 370 bp downstream of the *ARG8* ^*m*^ start codon, with 4 bp 3’ single-stranded complementary overhangs at the broken ends (Fig 4A). We previously reported that the galactose-inducible promoter regulating the expression of our mtLS-*Kpn*I was leaky, which stimulated the generation of deletion products prior to the addition of galactose [40]. In order to ensure that we did not have premature expression of *Kpn*I endonuclease, a temperature sensitive intein was inserted into the active site of *Kpn*I [44]. This intein self-cleaves at 20°C generating an active *Kpn*I enzyme. A control strain was also constructed utilizing a nonfunctional intein (int^dead^) that cannot self-cleave at any temperature [44].

This modification allows tight control of the activity of mtLS-*Kpn*I. Southern blot analysis shows no detectible digestion at the mitochondrial *Kpn*I recognition sequence, and no evidence of deletion product prior to induction of mtLS-*Kpn*I-int^ts^ (Fig 4B, lane P). *Kpn*I cleavage is achieved by inducing mtLS-*Kpn*I-int^ts^ expression with galactose and reducing the incubation temperature to 20°C (Fig 4B, lane 0). Furthermore, the frequency of DSB-induced DRMDs increases linearly with extended incubation time at 20°C prior to assaying at 30°C, suggesting that *Kpn*I activity is largely restricted to 20°C (S1 Fig). Longer incubations at 20°C generated a larger proportion of DSBs, but never exceeded 60% of the total *COX2* signal. This result may suggest that some genomes may not be accessible to the *Kpn*I enzyme or that alternative repair processes are rapidly repairing the break (S4 Fig). In subsequent experiments, 16–18 hours incubation at 20°C was used to ensure significant visible DSBs were generated.

Quantification of *COX2*-hybridizing species from independent experiments reveals that an average of 30.2 ± 16.7% of the *COX2* signal appears as a specific *Kpn*I digested fragment after 16–18 hours incubation at 20°C, and no measurable deletions are observed (Fig 4B and 4D). Two hours after cultures are returned to 30°C, deletion products can be observed, and their concentration continues to increase over the course of the experiment to represent, on average, 16.2% of the *COX2* signal (Fig 4B and 4D). Thus, the percent of the *COX2* signal that hybridizes to DRMD products after 8 hours at 30°C is approximately equivalent to two-thirds of the amount observed in the DSB at time 0. By 8 hours after shift to 30°C the cultures have reached saturation, and no further increase in deletion product was seen when cultures were monitored for an additional 4 hours (S4 Fig), suggesting that repair is complete by 8 hours in the wild-type strain. In addition, while breaks are stimulated at 20°C, the bulk of DRMD events occur after the cells are shifted back to 30°C, since deletions are not detected by Southern blot analysis at time of shift back to 30°C. In control cultures, expressing the mtLS-*Kpn*I-int^dead^, no stimulation of DRMD was observed, indicating that no significant cleavage of the int^dead^ occurs.

We included two additional probes in our Southern blot analysis to quantify potential changes in mtDNA content relative to nuclear DNA. The first, a mtDNA probe, hybridizes to the 21S rRNA gene approximately 10 kb from the site of the induced DSB. The second is a nuclear probe that hybridizes to the 25S rRNA gene. These probes were used to measure the ratio of mitochondrial to nuclear DNA during the course of these experiments. We find that shifting the cells to 20°C caused a slight decrease in mtDNA content relative to the nuclear control in both the mtLS-*Kpn*I::int^ts^ strain and mtLS-*Kpn*I::int^dead^ strain indicating that this decrease is due to the temperature shift and not the expression of active *Kpn*I (Fig 4C). The mtLS-*Kpn*I::int^ts^ expressing strains recovered significantly faster than the control strain, however, both the control and mtLS-*Kpn*I::int^ts^ strains fully recovered to pre-induction levels of mtDNA after eight hours of growth at 30°C (Fig 4C).

It has been reported that mitochondrial DNA can move to the nucleus, although this occurs rarely [45]. In order to ensure that the signal we are detecting in the Southern blots is originating from the mitochondrial genome, both pre- and post-induction wild type cultures were treated with ethidium bromide. Growth of yeast in culture medium in the presence of ethidium bromide has been demonstrated to suppress mtDNA replication [46]. Southern blot analysis confirms that greater than 98% of the signal from the *COX2* and the 21S rRNA probes is lost when the cells are subjected to ethidium bromide treatment. (S2 Fig).

**Fig 5:**
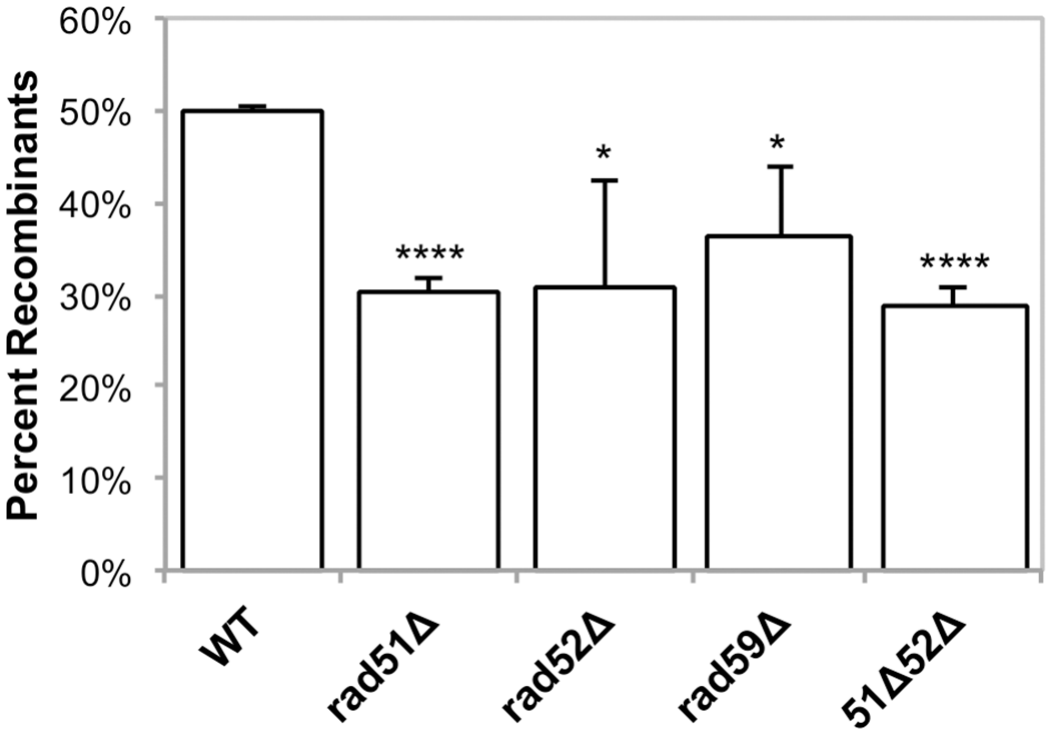
Induced mitochondrial repeat mediated deletions. Strains were grown in SRaffinose-Arg-Ura until OD_600_ = 0.150. Galactose was added to a final concentration of 2%, and the strains were shifted to 20°C for 16–18 hours in order to induce DSBs via mtLS-*Kpn*I. Appropriate dilutions were plated on YPD and allowed to grow for 3 days at 30°C. The colonies were then replica plated to YPG and SD-Arg. Growth on YPG was scored in order to determine the percent of colonies that had at least some cells that had undergone a deletion after non-selective growth. Error bars indicate SD. Asterisks indicate significant differences between the mutant and wild-type rates (* = *p* ≤ 0.05, **** = *p* ≤ 0.0001).

Eight hours after shifting to 30°C, approximately 16% of the mtDNA detected contains a deletion of the *ARG8* ^*m*^ reporter (Fig 4B and 4D). However, the distribution of the 16% of deleted genomes within the population of cells is unclear. For example, many deletions may be concentrated in a small subset of the cell population, or distributed throughout, with most cells containing one or more mitochondrial genomes with deletions. By assaying for respiring colonies, we can directly distinguish between these possibilities. Cells from time 0 in the above experiments (Fig 4B) were plated on non-selective medium, allowed to grow, and individual colonies were tested for the presence of the intact reporter (Arg^+^) or the deletion (respiration proficient) by replica plating. We found that 50% of the population is respiration competent, consistent with a wide distribution of mitochondrial genomes containing deletions (Fig 5). The majority of these colonies also contain Arg^+^ cells, indicating heteroplasmy at the time of plating. Significantly, we did not observe DSB stimulation of *ρ* ^-^ or *ρ* ^*0*^ (lacking mtDNA) *petites*, which would be both Arg^-^ and non-respiring. After cutting, 1.5% of the colonies from wild-type cells were both Arg^-^ and respiration deficient. This is comparable to the percentage of spontaneous *petites* seen in wild-type cultures prior to *Kpn*I-induced mtDNA DSBs, indicating the induction of the DSB does not lead to general mitochondrial genome instability (S3 Table).

### Rad51p, Rad52p, and Rad59p influence mitochondrial DSBR

To determine the effect of Rad51p, Rad52p, and Rad59p on the repair of mitochondrial DSBs, plasmids expressing either mtLS-*Kpn*I::int^ts^ (pEAS114) or mtLS-*Kpn*I::int^dead^ (pEAS115) were introduced into the mutant strains. Mitochondrial DSBs were induced as described above, and Southern blots reveal that the level of cutting by mtLS*Kpn*I-int^ts^ at the time of shift to 30°C in all of the mutant strains is equivalent to wild-type. (Table 1).

**Table 1:** Average level of cutting after 16 hours of mtLS*Kpn*I-int^ts^ induction.

	Average level of cutting	*p*-value^a^
Wild-type (EAS930)	30.2± 16.7%	-
*rad51-Δ* (ASY113)	31.5 ± 3.0%	0.90
*rad52-Δ* (ASY115)	40.6 ± 3.7%	0.21
*rad59-Δ* (ASY117)	24.9 ± 8.3%	0.52
*rad51-Δ rad52-Δ* (ASY119)	29.8± 2.0%	1.0

^a^ *p* values were calculated were calculated by un-paired, two-tailed t-tests.

In our genetic assay for induced deletions, 50% of colonies from wild-type cells had undergone at least one selectable deletion event (Fig 5), and each of the *RAD* single mutants exhibited a modest, yet significant, decrease in selectable deletion events when compared to wild-type. In the *rad51-*Δ, *rad52-*Δ, *rad59-*Δ strains 30% (*p* = 4.2 x 10^-5^), 31% (*p* = 0.03), and 36% (*p* = 0.03) of the cells were able to give rise to respiring colonies respectively, after 16–18 hours of inducing DSBs (Fig 4). As with the wild-type strain, non-respiring and Arg^-^ *petites* did not significantly increase after DSB induction in these strains (S3 Table).

**Fig 6:**
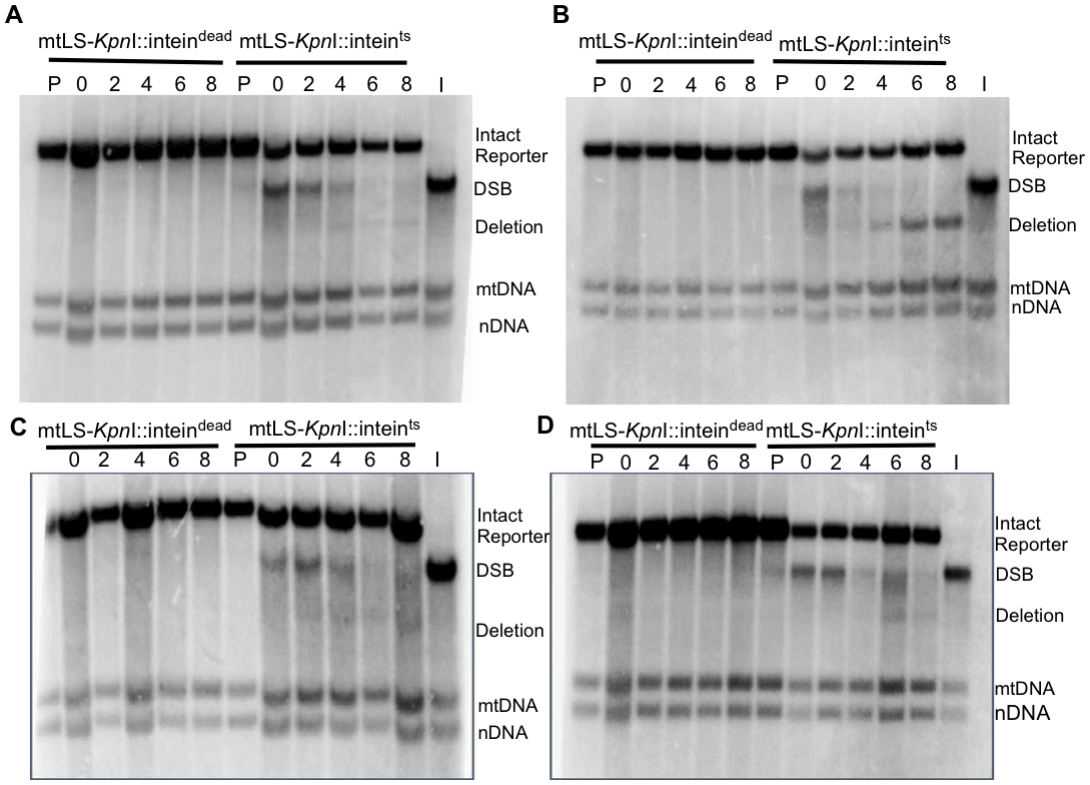
Repair of induced mitochondrial DSBs in HR single mutants. All samples were obtained after 16–18 hours of mtLS-*Kpn*I induction. DNA was extracted from the appropriate strains and digested with *Ava*II. Samples in lanes labeled P are from pre-induced cultures. Lane I is the t = 0 sample from the mtLS-*Kpn*I-intein^ts^ expressing strain digested with *Kpn*I and *Ava*II to demonstrate the migration of the reporter with a DSB in relation to the other *COX2* bands. (A) Representative Southern blot of *rad51-*Δ strains ASY114 (intein^dead^) and ASY113 (intein^ts^). (B) Representative Southern blot of *rad52-Δ* strains ASY116 (intein^dead^) and ASY115 (intein^ts^). (C) Representative Southern blot of *rad59-Δ* strains ASY118 (intein^dead^) and ASY117 (intein^ts^). (D) A representative Southern blot of *rad51-*Δ *rad52*-Δ strains, ASY120 (intein^dead^) and ASY119 (intein^ts^).

**Fig 7:**
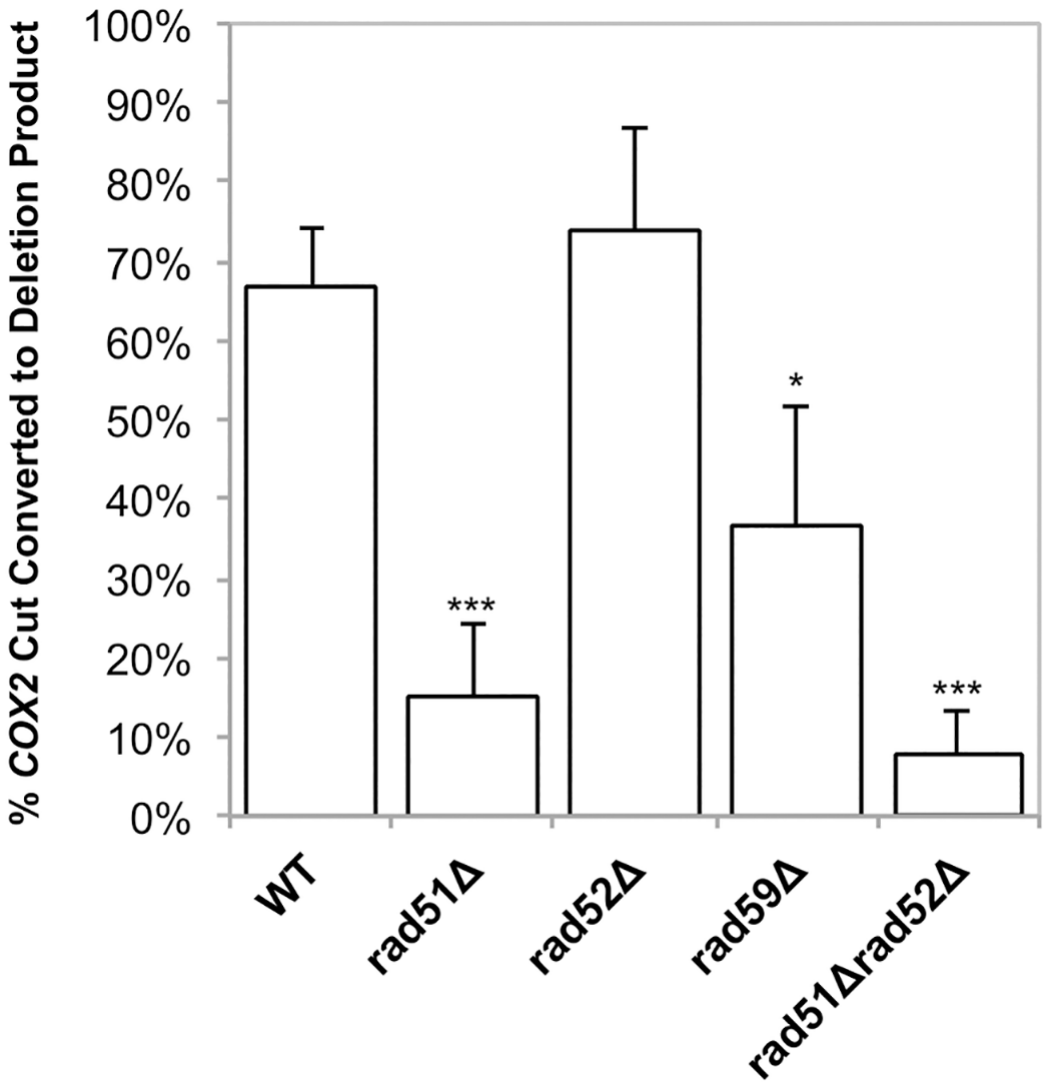
Conversion of DSBs to deletion product is impaired in HR mutants. Experiments were performed as in Fig 4. The ratios of the amount of *COX2* signal in the cut band at t = 0 and the amount of *COX2* signal in the deletion product at t = 8 were calculated. The intensity of the *COX2*-hybridizing bands was measured using Image Lab software (www.biorad.com). The proportions of the total *COX2* signal present in the DSB and deletion products were calculated as a percent of the total *COX2* signal. Error bars indicate SD. Asterisks indicate significant differences between the mutant and wild-type rates (* = *p* ≤ 0.05, *** = *p* ≤ 0.001).

Southern blots analysis of the mtDNA in *rad51-*Δ and *rad59-*Δ strains revealed that the same proportions of the mitochondrial genomes are being cut (31% and 25%, respectively) compared to the wild-type strain (Table 1). However, consistent with the genetic data, these cut genomes are not converted to deletion products to the same extent as wild-type (Figs 6A and 6C and 7). In the *rad51-*Δ strain after 8 hours of recovery only 14.4% ± 7.9 (*p* = 0.001) of the cut *COX2* signal had been converted to a deletion product compared to 66.8% ± 7.4 in the wild-type strain. In the *rad59*-Δ strain, 36.7% ± 14.9 (*p* = 0.01) of the cut *COX2* signal is converted to a deletion product. The cut band is completely lost in all mutant and wild-type strains after 8 hours of recovery, suggesting that DSB processing is not being eliminated but the pathway choice is altered in the absence of Rad51p or Rad59p.

Analysis of the *rad52*-Δ strain revealed no significant change in cut *COX2* signal at time 0, 40.6% ± 2 (*p* = 1.0) compared to wild-type (Table 1). Surprisingly, in contrast to the genetic data, the proportion of the cut *COX2* signal converted to deletion product, 55.9% ± 26.3 (*p* = 0.5) is equivalent to wild-type. We know that the distribution or maintenance of these genomes in the population of cells is not equivalent to wild-type because only 31% of the colonies contain respiring cells, while 50% of wild-type colonies contain respiration-competent cells after 3 days growth on solid medium.

The *rad51-*Δ *rad52-*Δ double mutant exhibited the same decrease in deletion events as the *rad51-*Δ and *rad52-*Δ single mutants, with 29% (*p* = 4.5 x 10^-5^) of colonies capable of respiration after induction of mtLS-*Kpn*I::int^ts^ (Fig 5). Interestingly, the mtDNA in this mutant displayed a similar significant decrease in the proportion of cut *COX2* signal converted to deletion product, 7.6% ± 5.7 (*p* = 0.0004), as the *rad51-*Δ strain (*p* = 0.3) (Figs 6D and 7). This would suggest that during the initial generation of deletion products after induction of a DSB that *RAD51* is epistatic to *RAD52* in the mitochondria.

We considered the possibility that the relative levels of the different *COX2*-hybridizing species in all cases might be affected by differences in growth rates of the mutant and wild-type strains under the conditions of the experiment. Monitoring the growth of the cultures by optical density and total cell counts throughout the protocol revealed that the *rad52-*Δ and *rad59-*Δ strains displayed a 20% increase in doubling time at 20°C, while the *rad51-*Δ mutant strain was comparable to the wild-type. All strains undergo one population doubling during the eight-hour recovery period. It is not likely then that the wild-type levels of deletion product generated in the *rad52-*Δ strain are dependent on this growth defect, considering the *rad59-*Δ strain also has inhibited growth at 20°C but clearly shows impaired deletion product formation. We believe there is no significant correlation of differences in growth rate with differences in DSBs or deletions (Fig 6B).

### Single-stranded DNA is present at break after induction of *Kpn*I

In the nucleus, the resection of the 5’ ends at a DSB commits the cell to repairing the lesion by HR. In order to determine if the DSBs detected by Southern blot in mitochondria are being processed, such that 3’ ssDNA is generated, the mtDNA was blotted and probed with a ssDNA probe that would anneal to 3’ single-stranded overhangs approximately 200 nucleotides from the site of the break. All of the strains exhibited a significant 3–4.5 fold increase in ssDNA after 16 hours of cleavage (Table 2). The increase in ssDNA at the site of a DSB suggests that the ends of mtDSBs are resected in a manner consistent with known nuclear HR models. The presence of similar levels of 3’ ssDNA in the wild-type, *rad51-*Δ, *rad52-*Δ, *rad59-*Δ, and *rad51-*Δ *rad52-*Δ strains is also consistent with HR models that show these proteins function in the repair of DSBs after resection has occurred (Table 2).

**Table 2:** Average fold change of amount of 3’ ssDNA at *Kpn*I cleavage site after induction.

Strain	Average fold change	*p*-value^a^
Wild-type (EAS930)	4.0	—
*rad51-Δ* (ASY113)	4.5	0.70
*rad52-Δ* (ASY115)	3.9	0.98
*rad59-Δ* (ASY117)	4.0	0.96
*rad51-Δ rad52-Δ* (ASY119)	3.0	0.54

^a^ *p* values were calculated were calculated by un-paired, two-tailed t-tests.

## Discussion

The faithful maintenance of the mitochondrial genome is required for eukaryotic cells to have a functional electron transport chain. Mutations and deletions in the mitochondrial genome can compromise energy production for the cell. However, studying mtDNA repair has been difficult, due to the necessity for intact mitochondrial genomes in most eukaryotic systems. Thus, the extent of mtDNA repair has remained unclear. For years it was believed that since there are multiple copies of the mitochondrial genome, the damaged mtDNA was simply degraded in lieu of repair [47]. While there is evidence that mitochondrial genomes are degraded under high levels of chronic damage, it is unclear if this is the response under more moderate damaging conditions [48].

Mitochondrial base excision repair has been more extensively studied [9,49,50], indicating that at least some highly conserved repair pathways exist in mitochondria. In addition, there is evidence for homologous recombination (HR) repair pathways in mitochondria of several different species including yeast, *Drosophila*, and mice [40,51,52]. In this study, we have shown that the HR proteins Rad51p and Rad59p localize to the matrix of the mitochondria. We have also demonstrated that Rad51p, Rad52p and Rad59p impact the rate of spontaneous mitochondrial DNA deletions and the repair of induced mitochondrial DSBs in yeast. This is the first study to demonstrate that Rad51p, Rad52p, and Rad59p impact the repair of specific mtDNA lesions.

In the present study, we have greatly improved our reporter system to introduce a maximum of a single DSB per mitochondrial genome to identify the proteins involved in the repair of these breaks. We had previously generated a plasmid expressing a galactose-inducible, mitochondrially-targeted *Kpn*I endonuclease that showed evidence of expression under uninduced conditions [40]. To add an additional level of control over endonuclease activity, we have inserted a temperature-sensitive intein into the active site of the *Kpn*I enzyme. This modified expression construct does not result in efficient cleavage of the mitochondrial genome, since we must incubate at the permissive temperature for 16–18 hours to observe digestion of approximately 30% of the mtDNA by Southern blot analysis (Fig 3). However, it is likely that a larger proportion of breaks are present, but these breaks are not detectible due to the end resection that occurs at the site of DSBs. We have demonstrated that 3’ single-stranded ends are present at the site of the break following cleavage. The heterogeneous nature of these intermediates would render them undetectable by our current Southern blot techniques. We would also be unable to detect any breaks that occurred and were subsequently ligated with fidelity. This system is an improvement over our previously published mtLS-*Kpn*I construct, as there is no molecular or genetic evidence of mtDNA cleavage prior to induction, nor is there selection for cleavage-resistant genomes in our cultures. In addition, this level of digestion is sufficient to observe DNA repair over the course of 8 hours after shifting back to the non-permissive temperature for intein cleavage.

It bears noting that deletion products do not appear after extensive incubation at the permissive temperature for intein cleavage, although they are clearly visible by 2 hours after shift back to the non-permissive temperature. It is unlikely that this delay simply results from slow repair, because no deletions are visible on a Southern blot after 24 hours of incubation at the permissive temperature (S1 Fig). Rather, this observation suggests that at least one step of the deletion process is inefficient or non-functional at 20°C. Deletion formation does not appear to be the only temperature-dependent process. We observed a slight decrease in relative mtDNA copy number upon shifting the cultures to 20°C. However, a decrease was also seen in our controls expressing mtLS-*Kpn*I-int^dead^, leading us to conclude that temperature shift alone is cause of this temporary decrease in mtDNA copy number (Fig 4C). Consistent with this hypothesis, both strains recover by 8 hours after shift back to 30°C. We conclude that in our system, repairable DSBs do not stimulate mtDNA loss from the population of cells. Our genetic assay supports this observation, as induction of a specific mitochondrial DSB does not stimulate an increase in the loss of functional mtDNA. Therefore, mechanisms exist in cells to maintain functional mtDNA. It is important to note that this selection cannot be applied at the level of respiratory function, or ATP production, as the genomes containing the intact DRMD reporter confer an Arg^+^ phenotype, and have active mitochondrial protein synthesis, but do not assemble a functional electron transport chain. In yeast, there is evidence for a mitochondrial G1-to-S phase checkpoint that is activated in response to the loss of mtDNA and not linked to respiratory capacity [53], suggesting that the cell has a mechanism for sensing mtDNA. Clearly, although mtDNA can be eliminated by treatment of cells with ethidium bromide and spontaneous *petite* mutants arise in yeast cultures [10] this should not be taken as evidence that mechanisms do not exist to maintain an intact genome.

Our results demonstrate that Rad51p, Rad52p, and Rad59p are involved in a portion of DSBR events. In the nucleus, SSA is the primary HR pathway that generates DSB-induced deletions between repeated sequences [26], and it seems likely to be an important pathway for spontaneous events at direct repeats as well. This mutagenic DSB repair pathway leads to the deletion of one of the repeats and any intervening sequence. In the nucleus, SSA is independent of Rad51p, but requires Rad52p and Rad59p [43]. Previous studies have shown [26,27], and this study confirms, that in the nucleus, deletion of *RAD51* leads to an increase in deletions generated by a Rad52p-dependent pathway, likely SSA (Fig 3B). In contrast, in mitochondria, the deletion of *RAD51* caused a significant decrease in the frequency of spontaneous deletions and those generated after inducing a DSB. Genetically, the contribution of Rad51p to the generation of DRMDs was equivalent to Rad52p and Rad59p (Figs 3D and 5). This would imply that both compartments use at least some of the same proteins to generate these deletions, but that the pathways these proteins are participating in are unique compared to the nucleus. In the absence of the full suite of nuclear HR proteins in the mitochondria we speculate that HR proteins, such as Rad51, have been co-opted to perform their HR functions in contexts that are not necessary in the nucleus.

In regard to the generation of spontaneous deletions, one possible explanation is that the initiating event that generates spontaneous mtDNA deletions is a replication error such as slippage, or a stalled fork that triggers template switching, and thus relies on a different suite of proteins, but is still dependent on the presence of homologous sequences and thus requires HR proteins, such as Rad51p. In our assay where we induced a DSB, an explanation for the increased dependence on Rad51p for deletion formation, is that the AT-rich mitochondrial genome requires further stabilization of the annealed intermediate and thus utilizes Rad51p uniquely in this manner. However, at this time, we cannot rule out the existence of a novel Rad51p dependent mechanism that generates deletions solely in the mitochondria.

The localization of Rad51p and Rad59p to mitochondria strongly suggests that the effects we observe on deletion product formation in the mutant strains are direct, for several reasons. First, we demonstrated that mitochondrial DSB repair and homology-dependent deletions were specifically affected, while mitochondrial genomes were stably maintained, as indicated by the maintenance of wild-type copy number and the lack of additional ρ^-^ in our mutant strains. In addition, the epistatic relationships between *RAD51*, *RAD52*, *RAD59* with respect to mitochondrial phenotypes were not the same as the nuclear phenotypes, most notably in the case of Rad51p, which had a similar impact on mitochondrial DRMD’s as both Rad52p and Rad59p. Finally, their action at DSBs is consistent with their known function. Identification of mitochondrial-specific factors that interact either physically or genetically with Rad51p, Rad52p, and Rad59p will help to elucidate the mitochondrial specific repair pathways or pathway components.

## Materials and Methods

### Media and strains

Media used in this study were previously described [39]. *S*. *cerevisiae* strains used in this study are listed in S1 Table and are isogenic with DFS188 except NPY66, which is derived from DFS160 [38,54]. Single-deletion strains were constructed by one-step gene replacement of the wild-type gene with a *kanMX* marker using standard protocols [55]. Strains containing two gene replacements were constructed by mating isogenic haploid strains, dissecting diploids, and screening by PCR for the desired genotypes. The spontaneous DRMD reporter strain (LKY196) was constructed previously [39]. Gene deletions in this strain were constructed by mating and dissection. The desired genotypes were screened for by PCR. These strains were then cured of their mtDNA by treatment with ethidium bromide, and cytoduced with the mitochondrial reporter from NPY66 [56].

Mitochondrially localized, V5-tagged mtLS-*Kpn*I was constructed by amplifying the bacterial *Kpn*I gene from pETRK [57], using a 5’ primer containing the mitochondrial localization signal of *COX4 5*’-GGATCCATGCTGAGCCTGCGCCAGAGCATTCGCTTTTTTAAACCGGCGACCCGCACCCTGTGCAGCAGCCGCTATCTGGTGATGGTGATGATGGATGTCTTTGATAAAGTTTATAG-3’ and 3’ primer 5’- TCTTTTCAATCCATAATAATTCAA-3’. The PCR product was cloned into pYES2.1 using the TOPO TA cloning kit (Invitrogen) then transformed into Top10F’ bacterial cells containing pACMK^+^, a plasmid that carries the *Kpn*I methylase [57]. Transformants were screened by digestion with restriction endonucleases *Bam*HI and *Xba*I. A positive clone was confirmed by sequencing and named pEAS101.

Mutant alleles of the *VMA*1 intein from yeast were obtained from C. Tan (University of Missouri) on pUGal4int^dead^ and pS5Gal4intein TS303 [44]. Inteins were inserted into the mtLS-*Kpn*I endonuclease gene by gap repair in pEAS101. The TS303 and intein-dead alleles were amplified from the above plasmid with 50 bp of *Kpn*I flanking sequence using the same forward primer 5’-CAATAGATCCCGCAACTGATGTAAATGATCCTAAAATGTGGCAAGCATTGtgctttgccaagggtaccaat-3’ and reverse primers 5’-CCATTATTTGAATCCCAATAATTTTTTTTCATAACTTGATGACGTCCACAattatggacgacaacctggttggc-3’ and 5’-CCATTATTTGAATCCCAATAATTTTTTTTCATAACTTGATGACGTCCACActgatggacgacaacctgcttggc-3’, respectively. pEAS101 was digested with *Zra*I and contransformed into EAS748 [38] with each of the amplified intein cassettes. The primers are designed to insert the intein just before the cysteine at position 198 of *Kpn*I. Transformants were selected on SD-Ura medium. Genomic DNA was collected from the transformants and plasmids were rescued by the transformation of *E*. *coli* Top10F’ cells and selection on LB+ampicillin. Plasmids containing the intein were screened by digestion with *BamH*I and *Bgl*I and confirmed by sequencing. pEAS114 contains mtLS-*Kpn*I+intein^ts303^. pEAS115 contained mtLS-*Kpn*I+intein^dead^.

### Mitochondrial isolation and protease protection assay

Mitochondria were isolated as previously described in Diekert *et al*., 2001 [58]. The protease protection assay was performed as previously described in Diekert *et al*., 2001 with the following modification. 200μg of isolated mitochondria were used in each protease protection assay for both the ASY092 (Rad51-HA) and ASY127 (Rad59-HA) strains. 100μg of isolated mitochondria were used in the protease protection assay for DFS188 (WT). Samples were subjected to western blot analysis on 10% SDS-PAGE gels. Antibodies used for detection were as follows: 1:2500 dilution of anti-HA-HRP (Santa Cruz Biotechnology), 1:4000 anti-Cytb2 (generous gift from Dr. Tom Fox), 1:4000 anti-Cit1 (generous gift from Dr. Tom Fox), and 1:25,000 anti-POR1 (Invitrogen).

### Chromatin immunoprecipitation

A 150 ml culture (RCY248) was grown to OD_600_ = 0.800 in YPD and crosslinked in 1% formaldehyde for 20 minutes at room temperature. Cells were pelleted and washed twice in 1X TBS. Pellets were resuspended in 1ml of lysis buffer (50 mM Hepes-KOH, pH7.5, 150 mM NaCl, 1 mM EDTA, 1% TritonX-100, 0.1% sodium deoxycholate, 0.5% SDS) and 0.4 g of glass beads. Cellular lysates were obtained by bead beating at 4°C for 2.5 minutes. The lysates were then sonicated to generate fragments between 400-700bp in size. Lysates were precleared by incubating with 20μl of Protein A/G magnetic beads (Thermo Scientific). One milliliter of lysate was incubated over night with either 1 μg of anti-Rad51 antibody (Abcam) or anti-V5 (mock) (Invitrogen) rocking at 4°C. Immune complexes were precipitated by incubating with 20 μl of Protein A/G magnetic beads (Thermo Scientific) for 1 hour at 4°C. Beads were washed twice with lysis buffer, once with LiCl buffer (, and twice with 1X TE. Immune complexes were eluted twice with 150μl of elution buffer (1% SDS, 0.1M NaHCO_3_). Crosslinks were reversed by adding 0.2M NaCl and heating overnight at 65°C. DNA was purified and equal amounts of DNA were analyzed by qPCR using SYBR green mix (Biorad) with the following primer pair: 5’-GGTAAATAGCAGCCTTATTATG-3’, 5’-CGATCTATCTAATTACAGTAAAGC-3’.

### Measuring nuclear and mitochondrial DRMDs and respiration loss

The rates of nuclear and mitochondrial spontaneous DRMDs and the average frequency of respiration loss were determined as previously described [39,40]. Fluctuation analyses consisted of 10–20 independent samples each. Rates were determined using the method of the median [59], and each experiment was performed a minimum of 3 times. Induced DRMDs were measured by growing strains to saturation in SRaffinose-Arg-Ura. These cultures were then diluted back to an OD_600_ ~ 0.08 and grown to OD_600_ of 0.15–0.20. A pre-induction sample was removed and an appropriate dilution was plated on YPD and incubated for 3 days at 30°C. A final concentration of 2% galactose was added to the remaining culture, which was then incubated at 20°C for 16–18 hours. A post-induction aliquot was then removed and the appropriate dilution was plated on YPD and incubated for 3 days at 30°C. All colonies were replica plated to YPG to score for deletion events, and SD-Arg to score for intact reporters. The average frequency and standard deviation of three or more independent experiments is shown. *P*-values were determined by un-paired, two-tailed t-tests using Microsoft Excel for Macintosh 2011.

### Southern blot analysis

DNA for Southern blot analysis was prepared from cells grown as stated above. A pre-induction sample was obtained prior to induction with 2% galactose and shifting to 20°C. After 16–18 hours of cutting, the cultures were shifted back to 30°C and samples were collected after 0, 2, 4, 6, and 8 hours. Total cellular DNA was extracted using standard procedures and transferred to Amersham Hybond-XL (GE Healthcare) nylon membranes following standard protocols. DNA was obtained from three independent experiments for each strain. Representative blots are shown in Figs 3, 5 and 6. The *COX2*, nuclear 25S rRNA, and mitochondrial 21S rRNA probes were constructed as previously described [40]. Quantification of Southern blots was performed using Image Lab software (www.biorad.com). The percentage of the *COX2* signal represented by deletion product and break product was calculated by measuring the intensity of each band and dividing by the total signal for all 3 *COX2*-hybridizing bands.

In order to confirm that the signal from the *COX2* probe was originating from the mtDNA the mitochondrial DNA from both pre-induction and time 0 cultures were diluted 1:1000 into minimal media containing 0.125mg of ethidium bromide and grown to saturation [46]. The saturated cultures were diluted 1:1000 into minimal media containing 0.125mg of ethidium bromide and grown to saturation. DNA was obtained from these cultures using standard protocol. The ethidium bromide treated cultures as well as DNA collected from these cultures prior to the ethidium bromide treatment were analyzed by Southern blot as described above.

### Slot blot analysis of 3’ ssDNA

Samples collected at time 0 were also subjected to slot blot analysis in order to detect 3’-ssDNA near the *Kpn*I cleavage site. Total cellular DNA was extracted using standard procedures. Samples were prepared as previously described [60] with the following modifications. Native genomic DNA (1μg) was prepared in 10X SSC and denatured samples (0.15μg) were prepared in 0.4N NaOH for 5 minutes. The samples were neutralized by the addition of 3M sodium acetate. Native and denatured samples were transferred to Amersham Hybond-XL (GE Healthcare) nylon membranes with a slot blot manifold following the manufacturers suggested protocol (Schleicher & Schuell). The DNA was UV cross-linked to the membrane. The membrane was prehybridized for a minimum of 1 hour in prehybridization solution (6X SSC, 5X Denhardt’s, 0.05% sodium pyrophosphate, 3 mg boiled fish sperm) at 37°C. The membrane was probed with a small synthetic end-labeled oligo (5’ CAGCAATACCAGCTGTGAAATCG 3’) overnight at 48°C. This probe anneals 213 nucleotides up stream of the *Kpn*I cleavage site. The membrane was washed in 6X SSC, 0.05% sodium pyrophosphate at room temperature. Quantification of slot blots was performed using Image J software (http://rsbweb.nih.gov/ij/). The native samples were normalized to the appropriate denatured control. *p*-values were determined by un-paired, two-tailed t-tests using Microsoft Excel for Macintosh 2011. Three independent experiments were performed for each strain.

## Supporting Information

S1 Fig Frequency of induced deletions is a function of time at 20°C. (A) The frequency of DRMD’s was measured after inducing mtLS-*Kpn*I^ts^ with 2% galactose and incubating for 2, 4, 6, 8, and 24 hours at 20°C or 30°C. (B) DNA was extracted from the appropriate strains and digested with *Ava*II. Lane I is the uninduced sample digested with *Ava*II and *Kpn*I *in vitro* to demonstrate the migration of the reporter with a DSB in relation to the other *COX2* bands.
(TIFF)

S2 Fig *COX2* signal is dependent on the presence of mtDNA. All samples labeled pre were obtained prior to the induction of mtLS-*Kpn*I^ts^. All samples labeled 0 were obtained after 16 hours of induction of mtLS-*Kpn*I^ts^. All samples labeled + EtBr were grown to saturation in ethidium bromide twice in order to remove the mtDNA. Lane I contains DNA from the wild-type t = 0 timepoint of the mtLS-*Kpn*I-intein^ts^-containing strain digested *in vitro* with *Kpn*I and *Ava*II to demonstrate the migration of DNA with a DSB.
(TIFF)

S3 Fig HA signal is specific to RAD51-HA and RAD59-HA strains. Immunoblot analysis of whole cell extracts and mitochondrial extracts from RAD51-HA, RAD59-HA tagged strains and wild-type untagged strain with HA antibody.
(TIF)

S4 Fig Percentage of detectable DSB maximized after 24 hours at 20°C. (A) Induction of DSBs after 24 hours at 20°C reaches ~60% of the total COX2 signal. (B) After induction of mitochondrial DSBs, the amount of COX2 signal found in the recombinant product does not increase after 8 hours of recovery at 30°C.
(TIF)

S1 Table List of strains used in this study. (DOCX)

S2 Table Spontaneous respiration loss frequency in the *rad* mutants. The median frequency of spontaneous respiration loss was determined for each strain using 10–20 independent cultures. The average median frequencies for at least three experiments are presented. Mutant frequencies are compared to wild-type using unpaired two-tailed t-tests, and *p*-values are provided.
(DOCX)

S3 Table The average difference in *petite* frequency following double-strand break induction. The samples analyzed in Fig 4 were also assessed for *petites* (Arg-, non-respiring colonies). For each experiment, we calculated the difference in the frequency of *petites* from the same culture before and after DSB induction. The average difference for all experiments is shown. The unpaired two-tailed t-tests were used to compare the frequencies for each strain before and after break induction, and *p*-values are presented.
(DOCX)
