# Supplementary figures and images for: Members of the RAD52 Epistasis Group Contribute to Mitochondrial Homologous Recombination and Double-Strand Break Repair in Saccharomyces cerevisiae
Source: PLoS Genet. 2015 Nov 5;11(11):e1005664. doi: 10.1371/journal.pgen.1005664 (PMC4634946; doi:10.1371/journal.pgen.1005664)

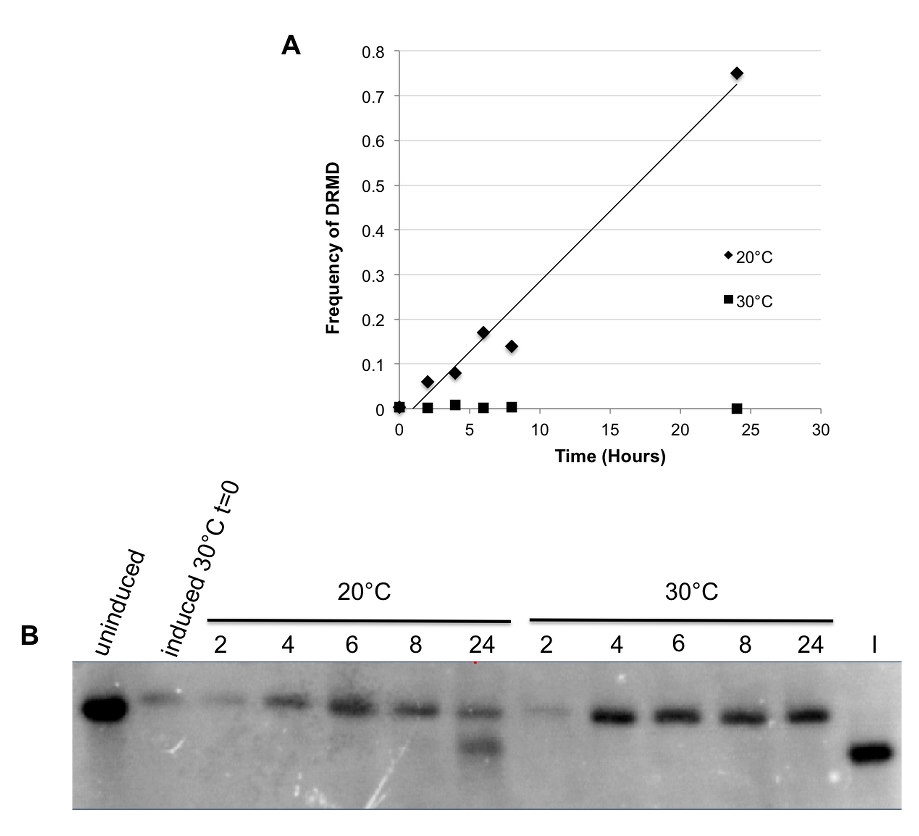

Supplement: S1 Fig — (A) The frequency of DRMD’s was measured after inducing mtLS-KpnIts with 2% galactose and incubating for 2, 4, 6, 8, and 24 hours at 20°C or 30°C. (B) DNA was extracted from the appropriate strains and digested with AvaII. Lane I is the uninduced sample digested with AvaII and KpnI in vitro to demonstrate the migration of the reporter with a DSB in relation to the other COX2 bands. (TIFF) [file pgen.1005664.s001.tiff]

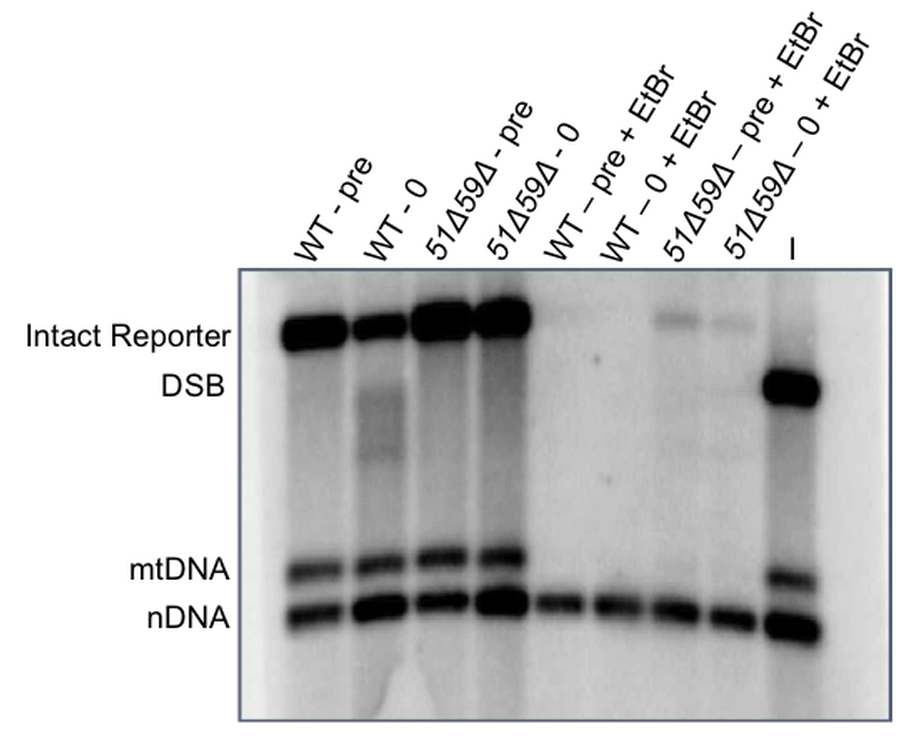

Supplement: S2 Fig — All samples labeled pre were obtained prior to the induction of mtLS-KpnIts. All samples labeled 0 were obtained after 16 hours of induction of mtLS-KpnIts. All samples labeled + EtBr were grown to saturation in ethidium bromide twice in order to remove the mtDNA. Lane I contains DNA from the wild-type t = 0 timepoint of the mtLS-KpnI-inteints-containing strain digested in vitro with KpnI and AvaII to demonstrate the migration of DNA with a DSB. (TIFF) [file pgen.1005664.s002.tiff]

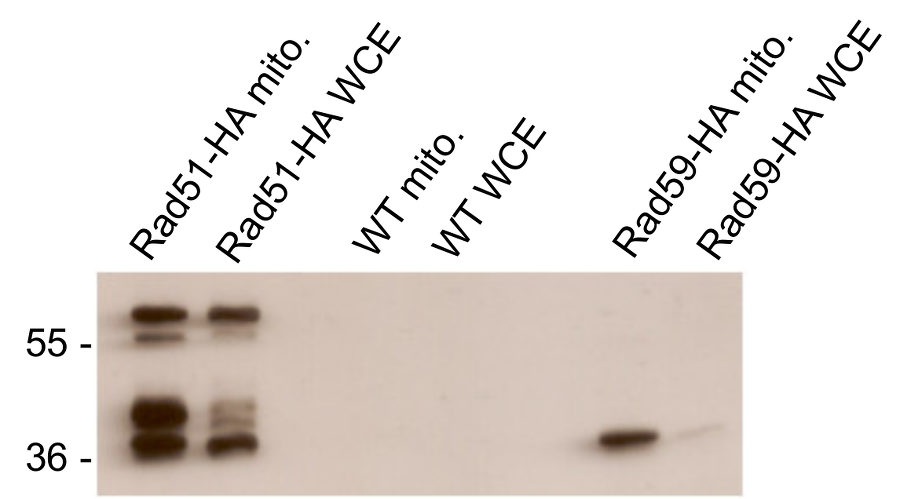

Supplement: S3 Fig — Immunoblot analysis of whole cell extracts and mitochondrial extracts from RAD51-HA, RAD59-HA tagged strains and wild-type untagged strain with HA antibody. (TIF) [file pgen.1005664.s003.tif]

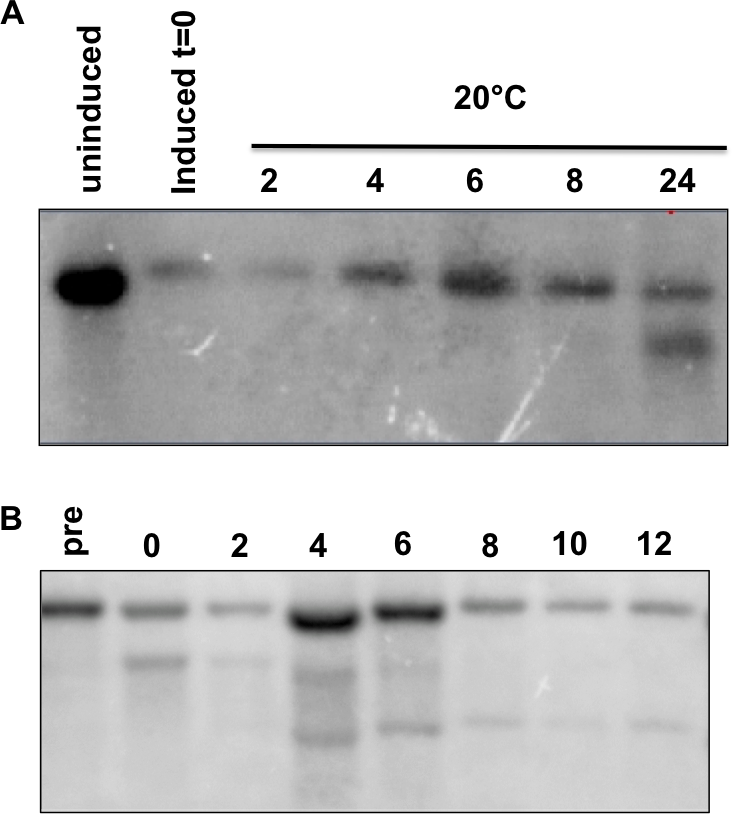

Supplement: S4 Fig — (A) Induction of DSBs after 24 hours at 20°C reaches ~60% of the total COX2 signal. (B) After induction of mitochondrial DSBs, the amount of COX2 signal found in the recombinant product does not increase after 8 hours of recovery at 30°C. (TIF) [file pgen.1005664.s004.tif]
