# Supplementary material for: Members of the RAD52 Epistasis Group Contribute to Mitochondrial Homologous Recombination and Double-Strand Break Repair in Saccharomyces cerevisiae
Source: PLoS Genet. 2015 Nov 5;11(11):e1005664. doi: 10.1371/journal.pgen.1005664 (PMC4634946; doi:10.1371/journal.pgen.1005664)
Supplement: S1 Table — (DOCX) [file pgen.1005664.s005.docx]

**Supplemental Table 1 Strains used in this study**

| **Strain** | **Relevant nuclear genotype** | **Mitochondrial genotype** | **Reference** |
| --- | --- | --- | --- |
| DFS188 | *Mat***a** *ura3-52 leu2-3, 112 lys2 his3 arg8::hisG* | ρ^+^ | [43] |
| LKY196 | *Mat***a** *ura3-52 leu2-3, 112 lys2 his3 arg8::hisG REP96::URA3::trp1* | mit^-^ *REP96*::*ARG8^m^*::*cox2* | [30] |
| EAS748 | DFS188 | mit^-^ *REP96*::*ARG8^m^*::*cox2* | [29] |
| NPY066 | *Mat*α *ade2-101 leu2Δ ura3-52 arg8-Δ*::*URA3 kar1-1* |  |  |
| LKY474 | LKY196 *rad51-Δ*::*kanMX* | mit^-^ *REP96*::*ARG8^m^*::*cox2* | This study |
| LKY402 | LKY196 *rad52-Δ*::*kanMX* | mit^-^ *REP96*::*ARG8^m^*::*cox2* | This study |
| LKY840 | LKY196 *rad59-Δ*::*kanMX* | mit^-^ *REP96*::*ARG8^m^*::*cox2* | This study |
| LKY1000 | LKY196 *rad51-Δ*::*kanMX rad52-Δ*::*kanMX* | mit^-^ *REP96*::*ARG8^m^*::*cox2* | This study |
| EAS930 | EAS748 + pEAS114 | mit^-^ *REP96*::*ARG8^m^*::*cox2* | This study |
| EAS972 | EAS748 + pEAS115 | mit^-^ *REP96*::*ARG8^m^*::*cox2* | This study |
| ASY092 | DFS188 RAD51-3xHA | ρ^+^ | This study |
| ASY113 | EAS930 *rad51-Δ*::*kanMX* | mit^-^ *REP96*::*ARG8^m^*::*cox2* | This study |
| ASY114 | EAS972 *rad51-Δ*::*kanMX* | mit^-^ *REP96*::*ARG8^m^*::*cox2* | This study |
| ASY115 | EAS930 *rad52-Δ*::*kanMX* | mit^-^ *REP96*::*ARG8^m^*::*cox2* | This study |
| ASY116 | EAS972 *rad52-Δ*::*kanMX* | mit^-^ *REP96*::*ARG8^m^*::*cox2* | This study |
| ASY117 | EAS930 *rad59-Δ*::*kanMX* | mit^-^ *REP96*::*ARG8^m^*::*cox2* | This study |
| ASY118 | EAS972 *rad59-Δ*::*kanMX* | mit^-^ *REP96*::*ARG8^m^*::*cox2* | This study |
| ASY119 | EAS930 *rad51-Δ*::*kanMX rad52-Δ*::*kanMX* | mit^-^ *REP96*::*ARG8^m^*::*cox2* | This study |
| ASY120 | EAS972 *rad51-Δ*::*kanMX* *rad52-Δ*::*kanMX* | mit^-^ *REP96*::*ARG8^m^*::*cox2* | This study |
| ASY127 | DFS188 RAD59-3xHA | ρ^+^ | This study |
| LKY463 | DFS188 *rad51-Δ*::*kanMX* | ρ^+^ | This study |
| RCY263 | DFS188 *rad52-Δ*::*kanMX* | ρ^+^ | This study |
| NPY121 | DFS188 *rad59-Δ*::*kanMX* | ρ^+^ | This study |
| NPY124 | DFS188 *rad51-Δ*::*kanMX rad52-Δ*::*kanMX* | ρ^+^ | This study |
| RCY248 | DFS188 ABF2-3xHA | ρ^+^ | This study |
