# Supplementary material for: Members of the RAD52 Epistasis Group Contribute to Mitochondrial Homologous Recombination and Double-Strand Break Repair in Saccharomyces cerevisiae
Source: PLoS Genet. 2015 Nov 5;11(11):e1005664. doi: 10.1371/journal.pgen.1005664 (PMC4634946; doi:10.1371/journal.pgen.1005664)
Supplement: S2 Table — The median frequency of spontaneous respiration loss was determined for each strain using 10–20 independent cultures. The average median frequencies for at least three experiments are presented. Mutant frequencies are compared to wild-type using unpaired two-tailed t-tests, and p-values are provided. (DOCX) [file pgen.1005664.s006.docx]

**Supplemental Table 2. Spontaneous respiration loss frequency in *rad* mutants**

|  | Average median frequency of respiration loss | |
| --- | --- | --- |
| Strains | **%** | *P*-value |
| Wild-type (DFS188) | 1.99 | -- |
| *rad51-Δ* (LKY463) | 2.11 | 0.83 |
| *rad52-Δ* (RCY263) | 1.56 | 0.45 |
| *rad59-Δ* (NPY121) | 0.89 | 0.09 |
| *rad51-Δ rad52-Δ* (NPY124) | 1.44 | 0.34 |
