# Supplementary material for: Members of the RAD52 Epistasis Group Contribute to Mitochondrial Homologous Recombination and Double-Strand Break Repair in Saccharomyces cerevisiae
Source: PLoS Genet. 2015 Nov 5;11(11):e1005664. doi: 10.1371/journal.pgen.1005664 (PMC4634946; doi:10.1371/journal.pgen.1005664)
Supplement: S3 Table — The samples analyzed in Fig 4 were also assessed for petites (Arg-, non-respiring colonies). For each experiment, we calculated the difference in the frequency of petites from the same culture before and after DSB induction. The average difference for all experiments is shown. The unpaired two-tailed t-tests were used to compare the frequencies for each strain before and after break induction, and p-values are presented. (DOCX) [file pgen.1005664.s007.docx]

**Supplemental Table 3. Average difference in *petite* frequency between pre- and post induction cultures**

|  |  | |
| --- | --- | --- |
|  | Avg. difference | *P-value* |
| Wild-type (EAS930) | 1.55% | 0.44 |
| *rad51-Δ* (ASY113) | 1.40% | 0.13 |
| *rad52-Δ* (ASY115) | 1.27% | 0.26 |
| *rad59-Δ* (ASY117) | 0.63% | 0.87 |
| *rad51-Δ rad52-Δ* (ASY119) | 1.98% | 0.82 |
|  |  |  |
